# Exploring the Triple Interaction between the Host Genome, the Epigenome, and the Gut Microbiome in Type 1 Diabetes

**DOI:** 10.3390/ijms22010125

**Published:** 2020-12-24

**Authors:** Duaa Ahmed Elhag, Manoj Kumar, Souhaila Al Khodor

**Affiliations:** Research Department, Sidra Medicine, Doha 26999, Qatar; delhag@sidra.org (D.A.E.); mkumar@sidra.org (M.K.)

**Keywords:** microbial dysbiosis, intestinal permeability, immuno-regulation, short-chain fatty acid, virome, single nucleotide polymorphism, *HLA*, NOD mice

## Abstract

Type 1 diabetes (T1D) is an auto-immune disorder characterized by a complex interaction between the host immune system and various environmental factors in genetically susceptible individuals. Genome-wide association studies (GWAS) identified different T1D risk and protection alleles, however, little is known about the environmental factors that can be linked to these alleles. Recent evidence indicated that, among those environmental factors, dysbiosis (imbalance) in the gut microbiota may play a role in the pathogenesis of T1D, affecting the integrity of the gut and leading to systemic inflammation and auto-destruction of the pancreatic β cells. Several studies have identified changes in the gut microbiome composition in humans and animal models comparing T1D subjects with controls. Those changes were characterized by a higher abundance of *Bacteroides* and a lower abundance of the butyrate-producing bacteria such as *Clostridium* clusters IV and XIVa. The mechanisms by which the dysbiotic bacteria and/or their metabolites interact with the genome and/or the epigenome of the host leading to destructive autoimmunity is still not clear. As T1D is a multifactorial disease, understanding the interaction between different environmental factors such as the gut microbiome, the genetic and the epigenetic determinants that are linked with the early appearance of autoantibodies can expand our knowledge about the disease pathogenesis. This review aims to provide insights into the interaction between the gut microbiome, susceptibility genes, epigenetic factors, and the immune system in the pathogenesis of T1D.

## 1. Introduction

Type 1 diabetes (T1D) is an auto-immune disorder caused by a complex interaction between the host immune system and different environmental factors in genetically predisposed individuals [1,2,3,4]. Furthermore, it is well known that T1D exhibit gender-related differences in which males are more predisposed to T1D in populations with the highest incidence, whereas a female bias was observed in the lowest risk populations (non-European origin), due to various factors [5,6,7,8].

According to the recent report from the International Diabetes Federation (IDF), a total of 600,900 children and adolescents up to 14 years old have T1D [9]. The incidence of T1D in children is increasing worldwide, with strong indications of a geographical-specific increase, with the highest rates of T1D (>5 per 100,000) found in North Africa and America [9].

Recently, a substantial increase in T1D incidence was observed [10], suggesting that multiple contributing factors must be involved in this higher incidence. Those factors include genetic and epigenetics contributors, autoimmunity, viral infections, antibiotics-mediated dysbiosis, gut microbiome composition, and lifestyle factors such as nutrition and modern diet [2,11,12,13,14,15]. Although certain *HLA* risk alleles are known to increase the susceptibility to T1D in children at risk, only 5% or fewer of them actually develop T1D [16], highlighting the importance of the non-genetic modifiers, in addition to other environmental factors in T1D pathogenesis [12,17,18]. While the genetic predisposition is considered to have a direct effect on the disease initiation, recent evidence indicated that different T1D risk alleles are also affecting the gut microbiome composition, however, it remains unclear how these risk alleles are interacting with the host gut microbiome and how these gut microbiomes affect the host epigenome leading to destructive autoimmunity [17,19]. The increasing incidence of T1D in western countries could be explained by the hygiene hypothesis, in which a lack of exposure to infectious agents can affect the maturation of the immune system [20]. 

While a great deal of progress has been made in our understanding of T1D pathogenesis, translating this knowledge into a clinical decision is still far from being achieved. This review aims to discuss the triple interaction between the host genome, the epigenome, and the gut microbiome in T1D.

## 2. Genetic Predisposition to T1D

The rapid evolution in genome-wide association studies (GWAS) along with the availability of large genomic consortia have transformed our ability to link between specific gene loci and their association with auto-immune diseases including T1D [21,22,23,24,25,26]. Recent GWAS studies highlighted that the risk for developing T1D is explained by the presence of certain Human leukocyte antigen class-II (*HLA* class II) risk alleles in addition to 60 plus non-*HLA* single nucleotide polymorphisms that have been recently identified [26]. These key genetic factors include the *HLA* alleles (mainly *HLA DR* and *DQ* genes) at position 6p21, which represent 30–50% of the T1D risk genes, in addition to *HLA I* and *HLA III* [21,27,28]. Around ~50% of the children carrying the *HLA-risk* genotypes *DR3/4-DQ8* or *DR4/DR4* develop T1D at a very young age (up to five years old), this risk increases when the child has a family history of T1D [21,27,29,30]. Interestingly, studies on familial T1D showed a higher incidence of T1D in offspring of fathers withT1D compared to mothers having T1D, in which the DR4-DQ8 haplotype was the most frequent haplotype in these children [31,32]. In addition to the *HLA* genes, 60 plus non-*HLA* risk alleles were shown to be involved in T1D pathogenesis, including several genetic variants in key immune genes such as the insulin gene (*INS*), the protein tyrosine phosphatase non-receptor type 22 gene (*PTPN-22*), the cytotoxic T-lymphocyte-associated protein 4 gene (*CTLA4*), the interleukin 2 receptor alpha (*IL-2RA*), and interferon-induced with helicase C domain 1 (*IFIH1*) in addition to *PXK/PDHB* and *PPIL2* genes [22,26,27,33,34,35,36]. Furthermore, growing evidence from the T1D twins studies showed concordance rates range between ~23–47% in monozygotic twins compared to ~3.8–16.4% in dizygotic twins which depends on the age at diagnosis [37,38,39,40,41]. It is also worth noting that these genetic factors do not provide a 100% positive predictive value, indicating that the progression into T1D is a complex interaction of both genetic determinants and other environmental factors such as the gut microbiome [17,42].

## 3. Gut Microbiome, Immunity, and T1D

Gut microbiota is the collection of microorganisms living in the gastrointestinal tract including bacteria, viruses, fungi, protozoa, archaea, and accounting for 500–1000 different species, which in healthy individuals, is predominated by two major bacterial phyla *Bacteroidetes* and *Firmicutes* [43,44,45]. The benefits of these microbes range from vitamin synthesis, energy homeostasis, maturation of the immune system among others [44,46,47]. Besides, the byproducts of the gut microbiota can modulate the host physiology and metabolism by facilitating digestion and extraction of energy from indigestible substrates such as extraction of short-chain fatty acids (SCFAs) from indigestible fibers [44,46]. SCFAs are used as an energy source by the intestinal mucosa, which can in turn maintain the intestinal homeostasis by regulation of the immune response and tumorigenesis in the gut [47].

Different factors affect the composition of the gut microbiota including the mode of delivery, diet, lifestyle, sex hormones, genetic background, pharmaceutical agents, use of antibiotics, and even the pH of the drinking water [48,49]. The alteration in the gut microbial composition has been involved in the pathogenesis of a wide array of diseases such as cardiovascular disease, gastrointestinal disease, and metabolic disorders including T1D, T2D, and obesity among others [50,51,52,53]. Interestingly, many studies have compared the microbiome composition between individuals with T1D (or those having a genetic risk of T1D) and healthy controls, in which they found differences in the gut microbiome composition, suggesting a role of the dysbiotic gut microbiome in disease pathogenesis [54,55,56,57,58,59].

### 3.1. Mode of Delivery and Risk of T1D

The mode of delivery is known to affect the gut microbiome composition, as the normal colonization of the infant’s gut is usually mediated through vaginal delivery in which the infants born through the vaginal canal are exposed to the vaginal microbiota (mainly *Lactobacillus*, *Prevotella*, *Bacteroides*, *Escherichia*, *Shigella*, and *Bifidobacterium*) [60]. In contrast, Cesarean sections (C-section) were shown to be associated with delayed acquisition of the vaginal microbiota (such as *Bacteroides* species), which is linked with the lower levels of Th-1-associated chemokines CXCL10 and CXCL11 in the infants’ blood, suggesting that these microbes are essential to enhance the production of cytokines necessary for neonatal immunity [61,62].

Growing evidence indicates an association between the elective C-Section and the increased risk of T1D compared to vaginal delivery, exhibiting up to 20% increased T1D risk at a younger age, which cannot be explained by other known confounders [62,63,64,65,66,67]. The association between delivery via a C-Section and the decreased levels of different microbial metabolites such as riboflavin and folate were recently highlighted, suggesting that the impaired folate biosynthesis might be linked to the defective immune functions of natural killers against viral infections and this can play a possible role in triggering the onset of T1D [64,68,69]. Furthermore, this may be explained by the lack of exposure to the beneficial microbiota of the mother during C-section, in addition to the delayed start of breastmilk lactation which is necessary for the growth of healthy intestinal microbes in the infant’s gut [62]. Furthermore, using broad-spectrum antibiotics during the first two years of life is associated with an increased rate of T1D in children born by either intrapartum C-section or pre-labor C-section, compared to vaginally delivered children [70]. Overall, these studies indicate the important role of a healthy microbiome even during early life to maintain a healthy immune system.

### 3.2. Environmental Factors, Gut Bacteriome, and T1D

**Figure 1:**
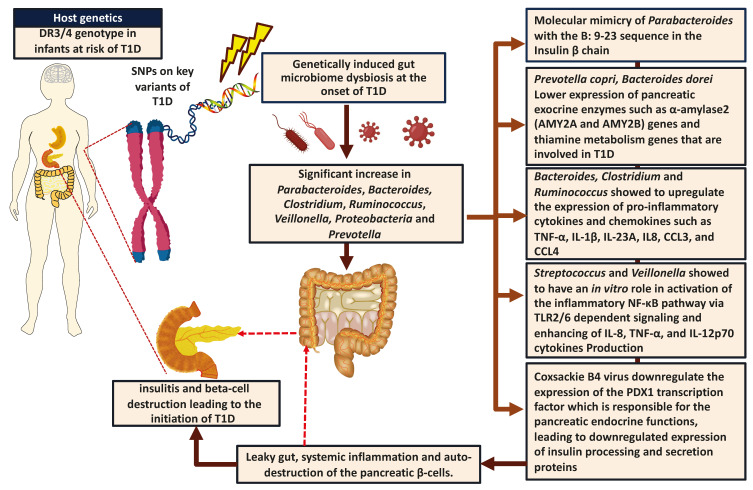
Interaction between host genetic, the gut microbiome in individuals with T1D. T1D is well known to be associated with microbial dysbiosis, a leaky gut, and intestinal inflammation. Changes in the gut microbial composition can enhance different pathological mechanisms in which each microbiome shows a different role, affecting the expression of the pancreatic exocrine enzymes such as α-amylase2 (*AMY2A* and *AMY2B*) genes as well as the thiamine metabolism genes known to be involved in T1D. The figure was created using BioRender.com and Science slides software.

The TEDDY study (The Environmental Determinants of Diabetes in the Young) was initiated to identify the changes in gut microbial composition in young children with risk to develop T1D and link them to the onset of T1D as shown in Table 1. Although these studies observed ethnicity-dependent differences in the microbial composition, they also observed an overall significantly higher abundance of *Parabacteroides* even at the onset of T1D in children [3,58,71,72]. Additionally, a higher abundance of *Bacteroides*, *Streptococcus*, and *Lactococcus*, and lower levels of *Bifidobacterium*, *Akkermansia*, and *Ruminococcus* were observed in the European T1D children [3,58]. Comparative gut microbial analysis of individuals with or without T1D revealed a significant decrease in the abundance of *Bifidobacterium* and *Lactobacillus*, and a significant increase in *Clostridium*, *Bacteroides*, *Ruminococcus*, and *Veillonella* species either in auto-antibody positive individuals or in T1D subjects which showed to be associated with the poor glycemic control and the enhanced intestinal permeability as shown in Table 1 [54,55,56,57,59,73] and Figure 1. This dysbiotic gut microbiome can contribute to the pathogenesis of T1D via multiple mechanisms. In the abundance of Gram-negative bacteria in the gut, lipopolysaccharide (LPS) is shown to modulate the intestinal epithelial barrier, affecting the inflammatory response in T1D subjects by upregulating the mRNA expression of various inflammatory cytokines including IL-1β, IL-18, and IL-12 in addition to the enhanced expression of CD80 co-stimulatory molecule [74]. Furthermore, *Clostridium*, *Bacteroides*, *Ruminococcus*, and *Veillonella* were shown to be linked with an in vitro higher expression of various inflammatory cytokines and chemokines such as TNF-α, IL-1β, IL-23A, IL-6, IL-8, CCL3, and CCL4, in which the translocation of these bacteria from the gut into the blood circulation might be associated with a leaky gut epithelium and enhanced intestinal immune infiltration in T1D subjects, leading to intestinal inflammation and autoimmunity [55,57,59,71,72,75,76] as shown in Table 1. This was also combined with a lower abundance of beneficial SCFA producing bacteria (*Bifidobacterium* and *Lactobacillus)* that may play a role in maintaining the integrity of the gut and lowering the auto-immune response as shown in Table 1 [54,55,56,74,76,77].

Regarding the role of diet and its effects on the gut microbiome composition in genetically susceptible T1D infants, the BABYDIET study showed an association between higher levels of *Bacteroides*, early non-milk complex diet introduction with an increased risk for early autoantibody development [95]. This could be mediated by the lower abundance of butyrate-producing bacteria which may impair the gut integrity allowing the translocation of *Bacteroides* and other food antigens into the blood circulation, enhancing the production of inflammatory cytokines and leading to systemic inflammation [95]. Furthermore, the recent Diabetes Prediction and Prevention study (DIPP) studies observed an increased abundance of *Bacteroides doeri* and a lower abundance of *Clostridium* clusters IV and XIV in T1D subjects [73,96,97]. Also, an increase in the abundance of *Proteobacteria* and *Gammaproteobacteria* (normally a pathobiome), was observed in T1D subjects with the genotype *HLA-DQ8*, *-DQ2* or both, which was possibly partly due to low fiber and high-fat diet [86,98,99] as shown in Table 1.

Interestingly, the T1D TrialNet Natural History Study assessed the gut microbiome changes that occur before and after the seroconversion period (when β-cells or insulin autoantibodies are produced) [100]. In this study, the canonical discriminant analysis showed similar microbiome clustering in seropositive and seronegative first-degree relative T1D subjects and this composition was distinct from those new-onset individuals and unrelated healthy control [100]. Although there was similar microbiome clustering in the two seroconverters groups, this study showed a significant increase in *Bacteroides* (well known as diabetogenic) in the seropositive subjects compared to the seronegative group, indicating that changes in the gut microbiome are strongly associated with islets auto-immunity [100]. Another study conducted in children with risk for developing T1D revealed an important correlation between the increased intestinal permeability and systemic inflammation [84]. This was linked with the increased abundance of gut *Proteobacteria* [84]. LPS from *Proteobacteria* (mainly *E. coli*) can mediate inflammation and endotoxemia in the gut through activating the NF-κB signaling pathway leading to enhanced expression of pro-inflammatory cytokines such as TNFα, IL-1β, IL-6, hence affecting the integrity of intestinal epithelia [86,87,88]. Furthermore, LPS from different diabetogenic bacterial species exhibit a variation in their immunogenicity, which may play a role in mediating the autoimmune response [101,102]. For example, LPS from *E. coli* showed to enhance a stronger inflammatory response in human PBMCs and dendritic cells leading to a higher production of inflammatory cytokines when compared to LPS from *B. dorei* [101,103]. This might be due to the difference in the lipid A structure between the two bacterial species [101,104,105]. Interestingly, LPS from *E. coli*, but not from *B. dorei* can mediate endotoxin tolerance in NOD mice, lowering the incidence of diabetes in them [101].

The DIABIMMUNE study aimed to study the host-microbe immune interactions and their effects on autoimmunity, in which they observed a higher proportion of *Bacteroides* species in Finnish and Estonian infants leading to a higher incidence of T1D compared to Russian infants [101]. This could be explained by the competitive effects between *Bacteroides* species and *E. coli*, in which *B. dorei* showed to inhibit the endotoxin tolerance to *E. coli* LPS in human cells, enhancing the inflammatory response [101]. This was consistent with another study that indicated the immunoinhibitory properties of *Bacteroides* LPS, making it less effective in providing early life signals required to maintain mucosal homeostasis (and prevent inflammation) than other forms of LPS [101,103]. Interestingly, it is well known that the balance between the activation or suppression of the M1 or M2 macrophages can either enhance or terminate the auto-immune response in the gut which can be affected by the gut microbiome [106]. The low-grade intestinal inflammation in individuals with T1D was shown to enhance the polarization of the pancreatic macrophages into the M1 classically activated macrophages [107], in which the bacterial LPS acts as an activation signal affecting the classically activated M1 macrophages by interacting with its Toll-like receptor 4 (TLR4) receptor and inducing the phosphorylation of both STAT1α and STAT1β [108]. Furthermore, LPS affects the expression of the inflammatory genes by downregulating the expression of the (*P2Y(2)R*) G-protein-coupled which is necessary for TLR4 phosphorylation in macrophages, mediating the production of the pro-inflammatory cytokines and the type I interferon (IFN) [80]. These cytokines are involved in organizing the trafficking of autoreactive CD8^+^ T cells towards the islets which are associated with the initiation of T1D [88,106,108,109,110,111]. The dynamic role of the microbiota in modulating the immune system is well known [46,47]. However, its unique mimicry to the human peptides and their interaction with the host autoantigen has been recently assessed, a study revealed a molecular mimicry between 17 gut bacterial antigens in *Parabacteroides distasonis* and the insulin B-chain peptide B: 9–23 (key epitope in T1D) [112]. This could be explained by the presence of a sequence in hypoxanthine phospho-ribosyltransferase in *P. distasonis* similar to the 9–23 sequence of the insulin β chain [82,113,114] in which this mimicking protein can activate T lymphocytes against pancreatic β cells when binding to the T cell receptor (TCR) [82]. This microbial interaction with the host peptide is considered one of the most important developments in the field of T1D and will provide a wealth of opportunities to explore microbial-based therapies for T1D subjects [113,114]. Interestingly, a recent meta-proteomics study showed the association between human fecal proteins and gut microbiome, linking them to the risk of T1D, in which the increased intestinal inflammation was associated with the significantly variant levels of adhesion molecules including CDHR5, CDH1, MUC2, FCGBP, and CEACAM5 in addition to the brush border enzymes MGAM and NAALADL1, which were shown to be associated with *Prevotella* and *Alistipes* [115]. This reflects a reduced output of exocrine enzymes which begins in islet autoantibody–positive individuals till the onset of T1D [115]. Furthermore, these proteins are known to maintain the intestinal barrier functions, acting as modulators of the immune system in which the variation in their concentration was shown to be combined with a lower abundance of anti-inflammatory bacterial species [115,116,117,118].

### 3.3. Gut Virome, Immunity, and T1D

The gut virome is known to be associated with the initiation of T1D, in which many in vitro and in vivo studies identified a positive correlation between the acute viral infection and the development of T1D [119,120,121,122,123,124,125,126,127,128,129]. T1D-associated viruses include *enteroviruses* such as *coxsackievirus B4* (*CV-B4*) which is known to mediate a low-grade pancreatic inflammation by enhancing viral replication, affecting the expression of β cell differentiation proteins, and lowering the expression of mitochondrial antiviral signaling proteins leading to persistent infection [130]. *CV-B4* is also known to lower the expression of *HLA* class I molecules at the surface of the infected pancreatic beta cells which are then targeted by the activated NK cells leading to cytolysis [120,130,131]. A recent report from the TEDDY study indicated that young children with a genetic risk for T1D experienced a prolonged *enteroviruses type B* and *adenovirus* infections that precede the development of T1D [120,121,130,132,133,134]. *CVB* viruses were shown to enhance the expression of IFN-γ, IL1-1B, IL15, and ICAM-1, which can activate the mononuclear cells against the pancreatic islets and upregulate TLR9 pathways leading to pancreatic cell destruction [120,130,132,133,134,135]. Moreover, many studies showed molecular mimicry between *rotaviruses* and *enteroviruses* molecules and pancreatic cells epitopes, for example, the viral tyrosine phosphatase (IA-2) showed a similar sequence to the islet antigens causing cross-reactive immune responses against β cells [136,137,138,139]. Interestingly, two observational studies hypothesized that if *rotavirus* infection can enhance the progression into T1D, then the vaccination with this virus may lower the incidence of newly diagnosed individuals with T1D [140,141]. In this study, the authors showed that *Rotavirus* vaccination resulted in a significant decrease in the incidence of T1D mainly in children aged 0 to 4 years compared to non-vaccinated children [140,141]. Moreover, a lower abundance of *Circoviridae* viruses combined with a lower richness of *bacteriophage* families in individuals with T1D was reported [59,127]. These *bacteriophages* can modulate the abundance of the T1D-associated bacteriome (such as *B. dorei*) which could be linked to the increased risk of islet autoimmunity [59,127]. It was also reported that *Enterovirus* infections in pregnant women increase the risk of T1D in their offspring, while another study showed that pregnant women with T1D had a higher abundance of *Picobirnaviruses*, *Tobamoviruses*, and *Enterovirus B* types compared to controls [142,143,144,145]. Overall, these studies explained the possible role of gut virome in the initiation of T1D, in which identifying the intestinal virome composition from birth till the onset of autoimmunity can help us in understanding the etiology of T1D.

### 3.4. Gut Mycobiome and T1D

The role of the gut mycobiome in T1D has been addressed in fewer studies that reported a non-significant increase in yeast-like fungi (mainly *Candida albicans*) in children and adults with T1D [146,147]. However, this fungal signature was not associated with levels of HbA1C suggesting a limited role of the gut mycobiome in the initiation of T1D [146,147]. It is worth noting that, since most of the studies that assessed the association between T1D and the gut microbiome composition were performed in small cohorts, there is a pressing need to confirm these changes in bigger cohorts, focusing more on the role of the genetic makeup of the host and how it may interact with different immunological and gut microbiome associated factors to enhance the auto-immune response.

### 3.5. Role of the Gut Microbiome in Animal Models with T1D

Similar to human studies, animal studies have also advanced our knowledge of T1D. The recent developments of the high-throughput technologies and availability of the germ-free mice models have shed light on the interaction of the microbiota, as well as its mediator molecules with various host functions [148,149]. Studies in various mouse models indicated a difference in the gut microbiome composition when comparing mice with and without T1D [150,151,152]. Interestingly, the most widely studied animal models are the non-obese T1D mice (NOD), which spontaneously develop T1D, in addition to the streptozotocin-induced T1D mice and rats [153,154,155,156,157,158]. NOD mice are known to have different genetically mediated immunological abnormalities such as the decreased number of the functional T lymphocytes and natural killer cells, in addition to the enhanced production of polyclonal antibodies to T cell-dependent antigens making them more susceptible to develop autoimmunity and T1D [159,160]. Streptozotocin-induced T1D mice and rats showed an increased abundance of pathogenic bacteria such as *Ruminococcaceae, Shigella*, *Enterococcus*, *Bacteroidaceae*, and *Alcaligenaceae*, which may enhance insulitis through promoting the production of IL-6, IL-12, IL-17, and IFN-γ, and mediating the inflammatory response in the gut [151,153]. Additional evidence from germ-free NOD mice indicated that the specific pathogen-free (SPF) NOD mice are more susceptible to develop T1D as shown in Table 2 [73,151]. This was associated with a significant increase in *Firmicutes*, *Bacteroidetes*, *Ruminococcaceae*, *Proteobacteria*, *Akkermansia mucinophila*, and *Enterococcus* that preceded the auto-immune response in T1D in SPF NOD mice [150,151,152], which further supports the role of the gut microbiota in the development and progression of T1D [73,151]. It is expected that the lack of a healthy gut microbiota affects the regulation and the maturation of the immune system [74,90,106,161]. This, in turn, leads to a deficiency in the development of mucosal-associated lymphoid tissue, specifically plasma cells and CD4^+^ T cells, and in turn promotes a differentiation imbalance between Th1, Th17 (T-helper cells), and Treg cells, which is associated with a higher rate of insulitis [151] as shown in Figure 1.

**Figure 2:**
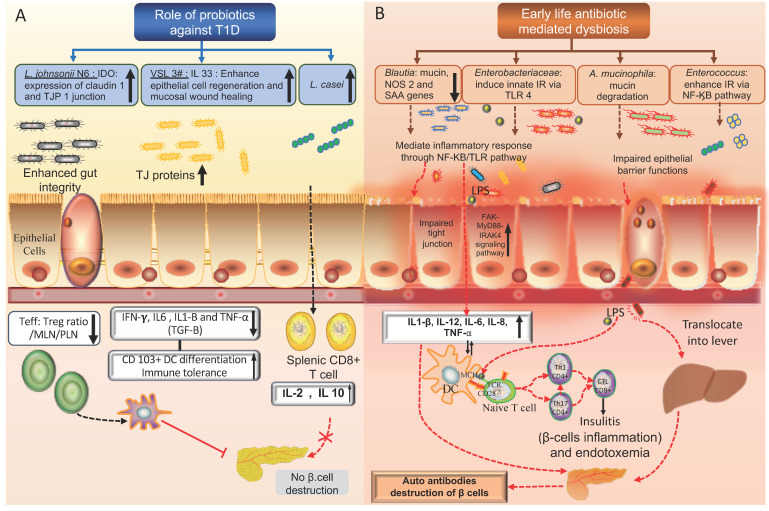
Comparison between the opposite effects of probiotics and early-life use of antibiotics in NOD mice. (**A**) T1D protective effects of probiotic bacteria in NOD mice: *Lactobacillus johnsonii N6* enhances the expression of INFγ and indolesamine 2,3-dioxygenase enzyme (IDO) which in turn increase the production of claudin-1 tight junction protein that maintains the integrity of the gut epithelium. *Lactobacillus casei* has probiotic properties, lowering the number of the splenic CD8^+^ cytotoxic T cells and promoting the expression of the anti-inflammatory cytokines (IL2, IL10) leading to increased immune tolerance in the gut. VSL3# probiotic has immune-modulatory functions, enhancing the expression of IL-33 cytokine that is necessary to maintain immune-tolerance in the gut and mesenchymal lymph nodes (MLN). (**B**) Role of antibiotic mediated dysbiosis in enhancing autoimmunity and pancreatic β-cells destruction in NOD mice: dysbiosis mediated by the use of antibiotics enhances the growth of pathogenic bacteria which promotes the inflammatory response via the TLR pathway which influences the gut permeability leading to metabolic endotoxemia. Endotoxemia stimulates the autoreactive T cells in the pancreatic lymph nodes leading to insulitis and β cell destruction. The figure was created using BioRender.com and Science slides software.

In contrast, it was also shown that the presence of a specific bacterial species such as *single-segmented filamentous bacterium* (SFB) or *Bacillus cereus* in the female NOD mice raised under germ-free conditions enhances the expression of the signature genes in Th17 cells such as *Il17a*, *Il17f*, *Il22*, *Il1r1*, and *Il23r* which may delay the onset of T1D through regulating the auto-immune response leading to enhanced gut integrity [150,165,166,167]. Moreover, comparative gut microbiota analysis between the NOD mice with and without T1D revealed a significantly higher abundance of four taxa that are linked to antibiotic-mediated dysbiosis, including *Enterococcus*, *Blautia*, *Enterobacteriaceae* species, and *A. mucinophila*, these taxa were shown to be associated with an accelerated progression into T1D (Figure 2) [156]. Growing evidence indicates that these bacteria can modulate the gut inflammatory response, either by increasing the gut permeability leading to metabolic endotoxemia or by enhancing the translocating of the LPS into the circulation which increases intestinal permeability through a TLR-4 dependent activation of FAK-MyD88-IRAK4 signaling pathway, leading to the development of insulitis and beta-cell destruction as shown in Figure 2 [154,168]. Furthermore, peptidoglycan isolated from *Firmicutes* and *Bacteroidetes species* in Akita T1D mice were shown to increase the risk of retinopathy in human retinal endothelial cells as shown in Table 2 [162]. While the absence of the myeloid differentiation innate immune adaptor gene (*MyD88*) in the NOD mice protects against T1D, it was observed that this protection is mediated by the gut microbiome, via enhancing the expression of the immuno-regulatory enzyme indoleamine 2,3-dioxygenase (IDO) in the pancreatic lymph nodes [161,169]. On the other hand, raising the *MyD88*-negative NOD mice under germ-free conditions quickly triggers the onset of T1D (Table 2) [161,169]. Moreover, a recent study revealed that raising of germ-free MyD88 knockout mice with a group of bacterial probiotics or *segmented filamentous bacteria* provides either partial or complete protection against T1D, highlighting the variability in the protection mechanism that is utilized by different bacterial species [161,170,171,172]. Progression into T1D in BDC2.5X TCR islet-specific transgenic NOD mice raised under a specific-pathogen-free environment was shown to be associated with an impaired gut barrier leading to microbiota-dependent endotoxemia, this endotoxemia can activate the migration of the islet autoreactive T cells to the pancreatic tissue leading to the initiation of T1D as shown in Table 2 [163]. In addition, the protective NOD mice such as the Idd3/5 and C57BL/6 mice, (which carry T1D protective alleles) have a distinct gut microbiome composition when compared to wild type NOD mice, showing differences in the relative abundance of *Lactobacillus*, *S24-7*, and *Ruminococcus* [17].

Although there are major differences between human and animal models in T1D associated gut microbiota, some commonalities emerge mainly in the mechanism by which different microbiomes can affect the regulatory functions of the immune system, mainly through the role of LPS and viral proteins which are known to be associated with the initiation of T1D [88,129,154].

## 4. Microbial Metabolites, Probiotics, and T1D

Intestinal microbes are well known to produce a range of molecules or metabolites, such as SCFAs including acetate (C2), propionate (C3), and butyrate (C4) that can modulate various host functions [173]. These molecules are byproducts for the bacterial fermentation of the dietary fibers in the colon known to maintain the gut epithelial integrity, enhance the colonic T-reg cells function, and to provide strong anti-inflammatory functions by modulating immune response [47,173,174]. Butyrate mediates anti-inflammatory functions in the intestinal mucosa through inhibition of the NF-κB transcription factor and activation of the *CX3CR1* of macrophages (Mfs) [90,91,174,175,176]. Intestinal butyrate is also involved in the regulation of TLR 4 gene expression, by reducing the LPS translocation and blocking of LPS stimulated dendritic cells (DC), in addition to enhancing the activity of Treg cells, as well as inhibiting immune response against the gut microbiota [90,91,174,175,176]. Moreover, butyrate is also reported to maintain the intestinal integrity by modulating the intestinal forkhead box protein P3 *FOXP3* (transcription factor responsible for activation of regulatory T-cell) [75,86,177]. Similar findings were documented in the mice model, for example, feeding NOD.Myd88^−/−^ with a diet rich in starch, fibers, or SCFA resulted in enhanced production of acetate and butyrate in their stool, hepatic, and peripheral blood, and protecting them against T1D [173,178,179,180]. This indicates the beneficial role of the microbial metabolites in providing immune- regulatory functions [173,178,179,180]. In addition, feeding of NOD mice with SCFA even at the onset of T1D gave them protection against T1D, by lowering the number of islets auto-reactive T cells and enhancing the proliferation of *FoxP3^+^* Treg cells in the gut mucosa, spleen, and pancreatic lymph nodes, thus enhancing immune tolerance [173,181].

On the other hand, the progression into T1D in TLR4-deficient (TLR4^−/−^) NOD mice showed to be associated with the gradual decrease of SCFA concentration in their portal blood vein [182]. This indicates the beneficial effects of microbial metabolites in maintaining a healthy gut and regulating immune response which shed the light on the potential therapeutic role of probiotics [183] many probiotic bacterial strains showed an immune regulatory function leading to protection against the autoantibody destruction of the pancreatic β cells [183]. Indeed, many microbial-based probiotics have been identified with potential benefit against T1D, for example, *VSL3#* which consists of eight beneficial *Lactobacillus* strains shown to prevent or delay T1D in NOD mice, this is mediated by releasing a number of molecules with anti-inflammatory activities, modulating the number of splenic CD8 T cells, enhancing the immune tolerance and the growth of beneficial bacteria in the gut [158,184,185] (Figure 2). Furthermore, probiotic species such as *Lactobacillus* spp. (*Lactobacillus. fermentum*) and *Clostridium* cluster IV and XIV spp., can promote the integrity of the gut epithelial barrier and protect against leaky gut through enhancing the expression of tight junction proteins including *ZO-*1, *Claudin-*1, *Occludin*, and *Cingulin*, protecting against T1D associated auto-immune response in the gut [186,187].

Due to the beneficial effects of probiotics and SCFA in maintaining the immuno-hemostasis, many clinical trials have been conducted in human subjects with T1D such as the TEDDY study, linking the early life consumption of probiotics and islet autoimmunity in genetically susceptible T1D children [188]. This study found a positive correlation between early life (the first 27 days of life), administration of probiotics (mainly *Lactobacillus* and *Bifidobacterium),* and the reduced risk of islet autoimmunity, especially in children with the highest risk [188]. Furthermore, a recent probiotic study showed that the administration of *Lactobacillus rhamnosus* GG can increase the serum tryptophan levels in kids with T1D, which in turn lowers the production of the inflammatory cytokines (IFN-γ, IL-17F) that is associated with the auto-immune response in PBMCs [189]. This suggests that prospective clinical studies are vital for the identification of potential novel microbiome-based therapeutic strategies for T1D.

## 5. Role of Genetic Predisposition on the Gut Microbiome Composition in Individuals with T1D 

Although GWAS studies have identified T1D associated genes including both *HLA* and non-*HLA* alleles, the predicted genetic contribution does not give us a 100% positive predictive value indicating the possible role of other environmental factors such as gut bacteriome and virome [17,190,191]. For example, the association between various SNPs in *PTPN22*, *PTPN2*, *IL10*, *IL2*, *IFIH1*, *INS*, *HLA-DRA*, and *CTLA4* genes and their impact on the gut microbiota composition has recently been revealed in individuals with autoimmunity and T1D [12,17,18,42,191]. Furthermore, rs2476601 and rs1893217 SNPs on *PTPN22* and *PTPN2* genes, respectively, were associated with a lower abundance of beneficial bacteria such as *Faecalibacterium*, *Bilophila*, and *Coprococcus*, in addition to a higher abundance of *Bacteroides* in many auto-immune diseases such as Crohn’s disease, and since these SNPs are common also in T1D, it may indicate a possible correlation with gut microbiome [192,193]. These genes are involved in the regulation of innate and adaptive immune responses against different viral and bacterial infections that are associated with the susceptibility to T1D [12,17,18,191]. Interestingly, several cohort studies have found an association between the *DR3/4* risk genotype (haplo-genotype A) and the higher abundance of *CV-B4* viral antibody levels, *Parabacteroides*, *Bacteroides*, *Clostridium*, *Ruminococcus Saccharimonadaceae*, *Klebisella*, *Veillonella*, *Akkermansia*, and *Erysipelotrichaceae*, in T1D children as shown in Figure 1 [30,42,72,191]. This could be explained by the fact that *DR3/DR4* risk alleles can increase the susceptibility to infection by making the immune system hyper-reactive [191]. Furthermore, non-*HLA* T1D associated SNPs can affect the immune system by lowering the activation and the signaling functions of T cells [194]. For example, *PTPN22* gene is involved in both T and B cell receptor signaling pathways in which SNPs in this gene can disturb the ability of T and B cells to recognize self from non-self-antigens [194]. In addition, SNPs on *PTPN2* are associated with an increased expression of pro-inflammatory cytokines in the intestinal epithelium, stimulating the activated Th1 and Th17 cells and impairing the function of regulatory T cells [192,194]. The stimulated Th1 and Th17 cells can mediate an inflammatory response in the pancreatic tissues, this was shown to be associated with molecular mimicry between the pancreatic cells and microbiome antigens mainly in *Bacteroides* and *Parabacteroides*. however, Further studies are required in order to identify the specific correlation between each SNP and its effect on the gut microbiome composition [192,194].

Interestingly, rs1990760 SNP on the *IFIH1* gene showed to be associated with variant levels of *Enterovirus* RNA in peripheral blood of children at risk for T1D [195,196]. It is well known that The *IFIH1* gene codes for the pattern recognition receptor MDA5 which is an innate immune receptor able to detect and interact against viral infection via activation of a cascade of antiviral responses including the stimulation of type I interferons and proinflammatory cytokines that showed to be associated with T1D [197].

As most of T1D related genes have been identified using advanced linkage studies and GWAS studies, genetic risk scores can be used for early prognosis of T1D combing early life factors such as diet, exposure to infections, and the measuring of early life auto-antibodies that appeared before the initiation of the disease, in which the combination of these factors with the associated gut microbial dysbiosis can expand our knowledge regarding the gut microbiome interaction network, by which this disease is initiated and the possible therapeutic targets that can be applied [21,23,24,27,198].

## 6. Role of Gut Microbiome in Gene Expression and Epigenetic Regulations of T1D

As discussed, the interactions between genetic predisposition and environmental factors are significantly associated with the initiation and development of T1D. One of the most important interactions is epigenetic regulation which includes histone modifications, DNA methylation, and non-coding RNA binding [19,199,200]. Those interactions can be triggered by the dysbiosis in the gut microbiome and their metabolites, these metabolites act as cofactors for the key epigenetic enzymes, affecting the methylation and acetylation, in addition to mediating variations in mi-RNA expression in different T1D related genes including *NF-KB P65*, *CTLA4*, *IL2*, and *FOXP3* [19,199,200].

### 6.1. Non-Coding RNA Binding

The non-coding RNA (ncRNA) are RNA transcripts that do not code for proteins in which the variation in their expression were shown to be associated with the risk of T1D [201]. This epigenetic role was observed mainly in the gut virome, in which a previous study revealed that *CV-B4* virus can mediate its pathogenicity to pancreatic cells by downregulating the expression of the PDX1 transcription factor which is responsible for the pancreatic endocrine functions, which in turn downregulate the expression of insulin processing and secretion proteins [130,202,203,204]. These effects showed to be mediated by modulating the expression pattern of miR-146a-5p and miR-23b in pancreatic ductal-like persistently infected cells [205]. Similarly, *CV-B4* virus has a suppressive role in some proteins that are associated with the pancreatic functions such as the mitochondrial metabolic proteins, CD63 and CD9, neurosecretory proteins (VGF), and secretogranins (SCG) that are responsible for the secretion of hormones and neurotransmission functions of the pancreatic cells [130].

### 6.2. Histones Modifications

Histones are proteins involved in DNA coiling to form the condensed chromatin in which some histone modifications including histone methylation, acetylation, and deacetylation are shown to be associated with the progression of T1D [206].

Recent comparative multi-omics analysis in individuals with T1D and healthy subjects revealed an association between the dysbiotic gut microbiota and the modulation of expression of different T1D related genes, linking the inhibitory effect of *Prevotella copri*, *B. dorei,* or *B. vulgatus* on the pancreatic exocrine enzymes such as α-amylase2 (*AMY2A* and *AMY2B*) genes and thiamine metabolism genes that are involved in T1D (Figure 1) [207]. In addition, dysbiotic gut microbiota (mainly G negative *Bacteroides)* may also induce an inflammatory response in the intestinal epithelium through the Myd88 signaling pathway, leading to higher gene expression of TLR 2, TLR 4, and TLR 9 in monocytes, and an increased expression of inflammatory cytokines, enhancing bacterial translocation which showed associated with the initiation of T1D [208,209]. Furthermore, the metabolites of beneficial gut microbiota such as intestinal SCFA (acetate and butyrate) can modulate histone deacetylase inhibitor (HDACi) function, mediating the decondensation and relaxation of chromatin, in which butyrate plays a protective role against T1D, by enhancing β-cells proliferation, leading to an enhanced gene expression of insulin in human and rat pancreatic β-cells [19,91,199,200,210,211]. This protective role of butyrate was confirmed in experimental T1D rats, showing a higher acetylation rate of H3, H4, and a downregulated expression of Pancreatic HMGB1 and NF-κB p65 proteins known to be involved in the pathogenesis of T1D by HDACi function [212,213,214]. In addition, SCFA (mainly butyrate) possesses an immunoregulatory function in different immune cells including T-reg cells, via mediating HDAC inhibition accompanied by histone H3 acetylation at the promoter of the *Foxp3* locus leading to enhanced Foxp3gene expression and providing protection against auto-immune response in individuals with T1D [91,175,180,199,215]. Interestingly, bacterial SCFA (mainly butyrate) possess an immunoregulatory function in different immune cells including macrophages which can also enhance the polarization of M2 macrophages through mediating the HDAC1 inhibition and histone H3K9 acetylation, promoting the STAT6 signaling pathways that are required for the M2 macrophage polarization, thus lowering the auto-immune response in the gut [216]. Furthermore, the epigenetic role of SCFAs in the intestinal T and B cells is mediated by enhancing the expression of the anti-inflammatory cytokine IL-10 by activating the *mTOR* complex (mammalian target of rapamycin complex), followed by an increase in glucose oxidation and production of acetyl-CoA, which acts as a cofactor for histone acetyltransferase enzymes (HATs), leading to anti-inflammatory effects in the gut, which protect against the auto-antibodies destruction of β-cells [184,199,215,217].

### 6.3. DNA Methylation

**Figure 3:**
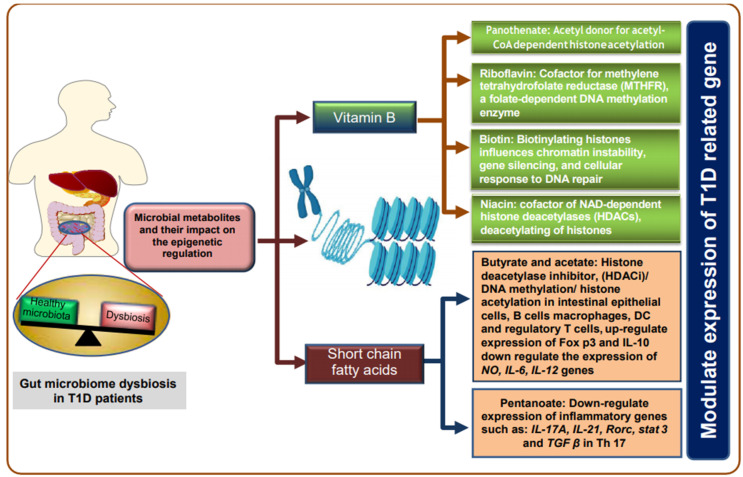
Role of microbial metabolites in the epigenetic regulation in T1D. Gut microbiota synthesize most of the B vitamins (Niacin, Biotin, Riboflavin, Pantothenate) that show different epigenetic roles and can modulate the expression of T1D associated genes. The therapeutic effects of SCFAs (mainly pentanoate) on autoimmune disorders are mediated by Th 17, macrophages, and dendritic cells (DC) via lowering the expression of inflammatory genes including *IL-6*, *IL-12*, *IL-17A*, *IL-21*, *Rorc* (RAR-related orphan receptor C gene), *Stat3* (Signal transducer and activator of transcription 3), *nitric oxide* (NO), lowering the immune response towards intestinal microbiota and enhancing the immune tolerance in the gut by T cell-independent IgA response. The figure was created using BioRender.com and Science slides software.

The gut microbiome can modify the DNA methylation patterns of host cells via the production of epigenetically active metabolites including folate, butyrate, and acetate that are necessary for DNA methylation [218]. Interestingly, therapeutic effects of SCFAs (mainly pentanoate) on autoimmune disorders were shown to be mediated by Th17, macrophage, and DC via lowering the expression of the inflammatory genes including *IL-6*, *IL-12*, *IL-17A*, *IL-21* [180,219], lowering the immune response towards intestinal microbiota and enhancing the immune tolerance in the gut by T cell-independent IgA response [220,221]. Furthermore, the gut microbiota can synthesize most of the B vitamins which have different epigenetic roles that are necessary to prevent nephropathy and improve renal function in individuals with T1D, however, dysbiosis in the gut microbiota can affect the concentration of these vitamins, altering methylation and histone modification status in immune cells, as shown in Figure 3 [19,200,222]. On the other hand, probiotic bacterial species such as *Bifidobacterium breve* and *L. rhamnosus GG* were also shown to downregulate the LPS-mediated expression of IL-17, IL-23, and CD40 via secretions of metabolites that promote the DNA methylation, thus maintaining the immune hemostasis in the gut which showed to be associated the reduced immune response [223].

## 7. Conclusions

This review summarizes the association between different genetic, epigenetic, and gut microbiome factors that together, can enhance the pathogenesis and progression of T1D. Although some complex interactions between the gut microbiome, the host genome, and epigenome in T1D have been revealed, still little is known about the effects of the host genome and different T1D variants on the gut microbiome, and whether these dysbiotic microbiomes are genetically determined. Further studies are required to elucidate the molecular mechanisms by which the microbial composition can contribute to protection from T1D, by understanding which bacterial species provide a specific beneficial protective role in T1D, and which type of metabolites can mediate this protective mechanism. Implementing multi-omics approaches will help to move towards identifying novel T1D initiating mechanisms and thus enable us to develop new therapies.

## Figures and Tables

**Table 1:** List of studies describing changes in the gut microbiome composition in individuals with T1D.

Study Details	Age and *HLA* Genotype	Changes in Gut Microbiome	Findings Related to T1D and the Possible Role of Gut Microbiome	Reference
16 cases 16 controls Caucasians	7.48 ± 0.87 years	-*Clostridium* ↑ -*Bacteroides* ↑ -*Veillonella* ↑ -*Actinobacteria* ↓ -*Firmicutes* ↓ -*Firmicutes to Bacteroidetes* ratio ↓ -*Lactobacillus* ↓ -*Bifidobacterium* ↓ -*Blautiacoccoides* ↓ -*Eubacteriumrectale group* ↓ -*Prevotella* ↓ which was linked with the higher glycemic level in children with T1D	-*Bacteroides and Clostridium* showed to upregulate the expression of pro-inflammatory cytokines and chemokines such as TNF-α, IL-1β, IL-23A, IL8, CCL3, and CCL4 -*Veillonella* showed to have an in-vitro role in the activation of the inflammatory NF-κB pathway via TLR2/6 dependent signaling pathway -Lower number of the beneficial bacteria (Lactic acid bacteria) that plays a role in limiting the inflammatory response in the gut through enhancing the expression of IL-10 and TNF-α and maintaining the gut integrity	[54,76,78,79,80]
15 cases 13 controls Caucasians	under 18 years old	-*Bacteroides* ↑ -*Ruminococcus* ↑ -*Veillonella* ↑ -*Blautia* ↑ -*Streptococcus* ↑ -*Bifidobacterium* ↓ -*Roseburia* ↓ -*Faecalibacterium* ↓ -*Lachnospira* ↓	-Lower bacterial diversity and higher gut permeability -*Bacteroides* and *Ruminococcus* showed to upregulate the expression of different inflammatory cytokines and chemokines such as IL-1β, IL-23A, CCL3, and CCL4 that are involved in the recruitment of immune cells in human islets leading to oxidative stress and insulitis -*Streptococcus* and *Veillonella* showed to have an in vitro role in the activation of the inflammatory NF-κB pathway via TLR2/6 dependent signaling and enhancing of IL-8, TNF-α, and IL-12p70 cytokines Production	[55,76,78]
20 cases 28 controls Caucasians and Afro-descendants	23.1 ± 8.6 years	-*Bacteroides vulgatus* ↑ -*Bacteroides rodentium* ↑ -*Prevotellacopri* ↑ -*Bacteroides xylanisolvens* ↑ -*Bifidobacterium* ↓ -*Lactobacillales* ↓	-Increase in the bacterial translocation through the epithelial barrier, leading to leaky gut, systemic inflammation, and destruction of the Pancreatic β cells -*Bacteroides vulgatus*, showed to activate the NF-κB inflammatory pathway in intestinal epithelial cells	[57,81]
903 non-Hispanic Children with T1D risk	3 to 46 months with *DQB1*, *DQA1* or *DQB1*, *DRB1* genotype positive	-*Parabacteriodes* ↑ -*Streptococcus* sp. ↑ -*Lactococcus* sp. ↑ Unclassified species ↓ *-Ruminococcaceae* ↓ -*Lactococcus* ↓ -*Streptococcus* ↓ -*Akkermansia* ↓	-Molecular mimicry of *Parabacteroides* with the B:9–23 sequence in insulin B chain could be linked to the initiating of T1D	[3,71,72,82]
11 cases T1D risk and 11 controls from Finland and Estonia	0 to 77 months with *HLA DR-DQ* genotype positive	-*Bacteroides ovatus* ↑ -*Bacteroides fragilis* ↑ -*Bacteroides vulgates* ↑ -*Lachnospiraceae* ↓ -*Veillonellaceae* ↓ -*Bifidobacterium* ↓ Viral changes: -*Circoviridae* viruses ↓ -*Microviridae* ↓ -*Myoviridae* ↓ -*Podoviridae* ↓	-*Bacteroides vulgatus,* showed to activate the NF-κB inflammatory pathway in intestinal epithelial cells -*Bacteroides fragilis* toxins (BFT) and colibactin cause DNA damage in the gut epithelial cells -Both are linked to leaky gut, systemic inflammation, and destruction of Pancreatic B cells -Bacteriophages changes that precede the seroconversion modulate the abundance of the dysbiotic T1D associated bacteria (mainly *Bacteroides)* suggesting the role of virome in triggering seroconversion	[59,81,83]
28 cases 27 controls from Finland, Estonia, France, Greece or Lithuania	1.3–4.6 years with *HLA DR-DQ* genotype positive	-*Streptococci* ↑ -*Bacteriodes* ↑ -non-butyrate-producing *Clostridium species* ↑ -butyrate-producing *Clostridium* clusters IV and XIVa ↓	-*Streptococci* and *Bacteriodes* produce glutamate decarboxylase, which may stimulate GAD autoimmunity via molecular mimicry -Lower butyrate production and higher gut permeability	[75]
10 cases 8 controls from Finland and Estonia	0–3 years with *HLA DR-DQ* genotype positive	-*Escherichia coli* ↑	-Releasing of LPS, DNA, and amyloid by bacteriophage infected *E. coli* may have a role in initiating autoimmunity -*E. coli* showed to have a role in DNA methylation, downregulating the expression of *CDKN2A* gene, which linked to the enhanced proinflammatory functions of CD14^+^ and CD16^+^ monocyte and the dysregulated functions of Treg cells in addition to the higher levels of HbA1C	[84,85]
73 cases 103 controls From Azerbaijan Jordan, Nigeria, and Sudan	3–19 years with *HLADQ8*, *DQ2,* or both genotype positive	-*E. coli* ↑ -*Eubacterium* ↓ -*Roseburia* ↓ -*Clostridia clusters IV or XIVa* ↓	-LPS from *Proteobacteria* enhances the inflammation and endotoxemia in the gut, acting as an activation signal for M1 macrophage through enhancing the NF-κB signaling pathways and upregulating the expression of pro-inflammatory cytokines such as TNFα, IL-1β, IL-6, affecting the integrity of intestinal epithelia, leading to autoimmunity and T1D	[86,87,88]
12 cases 10 controls (Han Chinese)	12–33 years	-*Bacteriodes/ Firmicutes ratio* ↑ -*Bilophila* ↑	-Higher HbA1c was associated with increased *Bacteriodes* -Higher number of anti-islet cell autoantibodies associated with decreased abundance of *Faecalibacterium* (butyrate-producing bacteria) and *Ruminococcaceae*	[86,89]
47 children with islet autoimmunity or T1D	5.3–16.3 years	-*Prevotella* ↓ -*Butyricimonas* ↓ -SCFA producing bacteria ↓ -bacterial diversity ↓	-Gut microbiome dysbiosis was accompanied by higher intestinal permeability. *Butyricimonas* and *Prevotella* species are butyrate-producing bacteria that showed to have immunomodulatory properties in the gut -Butyrate enhances histone H3 acetylation in the promoter of the *Foxp3* locus, promoting the differentiation of T_reg_ cells and regulating the balance between Treg and Th17 -Butyrate signaling via Gpr109a receptor showed to enhance the anti-inflammatory functions in colonic macrophages and dendritic cells, which in turn enhances the differentiation of Treg cells and the production of IL-10 and IL-18 in colonic epithelium, in addition to suppression of TNF α production in monocytes	[77,90,91,92,93,94]
41 controls	*HLA-DR* genotype positive			

HLA: Human leukocyte antigen; T1D: Type 1 diabetes; ↑: Increased; ↓: Decreased.

**Table 2:** Changes in gut microbial composition related to T1D in different animal models.

Animal Model Details	Changes in Gut Microbiome	Findings Related to T1D and the Possible Role of Gut Microbiome	Reference
SPF NOD mice with T1D	-Increased Ratio of G+/G− bacteria -α diversity ↓ -*Firmicutes* ↑ *-Bacteroidetes* ↑ -*Erysipelotrichaceae* ↑	-Deficiency in the development of mucosal-associated lymphoid tissue -imbalance between Th1, Th17, and Treg in the intestine and increased intestinal permeability	[150,151,152]
Streptozotocin-induced T1D rats	-Firmicutes/Bacteroidetes ratio ↑ -*Ruminococcaceae* ↑ -*Shigella* ↑ -*Enterococcus* ↑	-LPS enhances inflammatory response in the pancreatic β cells by upregulating the mRNA expression of various inflammatory cytokines including IL-1β, IL-18, and IL-12 in addition to enhanced expression of CD80 co-stimulatory molecule	[154]
Streptozotocin-induced T1D mice	-*Bacteroidaceae* ↑ -*Alcaligenaceae* ↑ -*Ruminococcaceae* ↑ -*Bifidobacteriaceae* ↑	-Increased expression of NOD1 and NOD2 genes in the pancreatic lymph node -Increased IL-6, IL-12, IL-17, and IFN- γ -invasive insulitis in the pancreatic islets	[155]
ACE2 knockout Akita mice with T1D	-*Firmicutes*/*Bacteroidetes* ratio ↑	Enhanced gut permeability, enhanced microbial translocation with higher amount of circulatory peptidoglycan which increases the risk of T1D associated retinopathy when transferred into HRECs via TLR 2 activation mechanism	[162]
SPF BDC2.5X NOD mice treated with DSS	-*Firmicutes* ↑ -*Deferribacteres* ↑ -*Porphyromonadaceae* ↑ -*Bacteroidetes* ↓ *-Prevotellaceae* ↓ -*Rikenellaceae* ↓	-Lower gut integrity -Decrease in expression of immune-regulatory mucins *Muc1* and *Muc3* -Increase in inflammatory cytokines in the intestinal mucosa (TNF-α, IL1-β, IL-23p19, IL-17)	[162,163]
Antibiotic induced dysbiosis in SPF NOD mice	-*Proteobacteria* ↑ -*Akkermansia mucinophila* ↑ *-Enterococcus* ↑ -*Blautia* ↑ -*Enterobacteriaceae sp* ↑ -*Clostridiales* ↓ -*Oscillospira* ↓ -*Ruminococcus* ↓	-Accelerated development of T1D -Lower numbers of RORγt^+^ Th 17 and FOXP3^+^ Treg Cells affecting the expression of genes that are associated with immunity and cholesterol synthesis -Increased intestinal permeability	[156,164]

T1D: Type 1 Diabetes; SPF: Specific Pathogen Free; NOD: Non-obese diabetic; ACE2: Angiotensin-converting enzyme 2; HRECs: Human retinal endothelial cells; TLR: Toll-Like Receptor; DSS: Dextran Sodium Sulfate; ↑: Increased; ↓: Decreased.

## Data Availability

Not applicable.

## Abbreviations

T1D	Type 1 diabetes
MHC-II	Major histocompatibility class II
SCFA	Short-chain fatty acid
Treg	T regulatory cells
TCR	T cell antigen receptor
IGRP-reactive CD8^+^ T cells	Islet-specific glucose-6-phosphatase catalytic subunit–related protein (IGRP) reactive CD8 T cell
LPS	Lipopolysaccharide
IAA	Insulin autoantibody
GADA	Glutamic acid decarboxylase antibody
FOXP3	Forkhead box P3
HDACi	Histone deactylase inhibitor
HMGB1	High-mobility group protein 1
NF-κB p65	Nuclear factor kappa-light-chain-enhancer of activated B cells
mTOR complex	Mammalian target of rapamycin complex
HATs	Histone acetyltransferases
PSA	Polysaccharide A
TEDDY	The Environmental Determinants of Diabetes in the Young
Rorc	RAR-related orphan receptor C gene
Stat3 transcription factor	Signal transducer and activator of transcription 3
TGF-β gene	Transforming growth factor β gene
TGF-β	Transforming growth factor β
ATRA	Alltrans retinoic acid
INS	Insulin gene 3
PTPN-22	Protein tyrosine phosphatase non-receptor type 22 gene
CTLA4	Cytotoxic T-lymphocyte-associated protein 4 gene
IL2RA	Interleukin 2 receptor alpha
IFIH1	Interferon-induced with helicase C domain 1
CDHR5	Cadherin-related family member 5
CDH1	Cadherin-1
FCGBP	IgGFc-binding protein
CEACAM5	Carcinoembryonic antigen-related cell adhesion molecule 5
MUC2	Mucin-2
MGAM	Maltase-glucoamylase
NAALADL1	N-acetylated alpha-linked acidic dipeptidase like 1
PXK	PX domain-containing protein kinase-like protein
PDHB	Pyruvate Dehydrogenase E1 Subunit Beta
PPIL2	Peptidylprolyl Isomerase Like 2
